# Precarity in the Modes of Living: Proposing an Index for Studying Health Inequities at the Ecological Level in Colombia

**DOI:** 10.3390/ijerph22040537

**Published:** 2025-04-01

**Authors:** Hugo-Alejandro Santa Ramírez, Andrés-Felipe Ramírez-Giraldo, Hugo Pilkington, Carme Borrell, Gabriel-Jaime Otálvaro-Castro

**Affiliations:** 1 Faculty of Medicine, Institute of Global Health, University of Geneva, 1202 Geneva, Switzerland; 2 Health Policies and Management Research Group, National Faculty of Public Health, Universidad de Antioquia, Medellín 050010, Colombia; 3 UMR7533 Ladyss, Département de Géographie, Université Paris 8 Vincennes-Saint-Denis, F-93526 Saint-Denis, France; 4 Agència de Salut Pública de Barcelona, 08023 Barcelona, Spain; 5 CIBER Epidemiología y Salud Pública (CIBERESP), 28029 Madrid, Spain; 6 Institut de Recerca Sant Pau (IR SANT PAU), 08041 Barcelona, Spain

**Keywords:** modes of living, precarity, deprivation, child mortality, social determination of health, Latin American social medicine, collective health

## Abstract

Deprivation indices are used to monitor health inequities. However, their theoretical underpinnings have been based on the context of Western industrialized countries, which have distinct social and historical backgrounds compared to Latin America and the Caribbean and countries in the Global South. Following the Latin American Social Determination of Health perspective, particularly the category Modes of Living supported by the construct of precarity, we aimed to develop an index of precarity in the modes of living at the department level in Colombia and assess its geographical distribution and potential value for public health. We conducted an ecological cross-sectional study with national administrative records. We developed a precarity index through Principal Component Analysis and performed spatial autocorrelation analyses and regression models with child mortality indicators. Our final index comprised twenty indicators representing four dimensions of the modes of living and power relations. We found precarity not to distribute randomly in Colombia, with a center-periphery divide and higher precarity observed in the country’s margin. We also found an association of our index with under-five mortality (SMR = 1.19; 95%CI 1.08–1.31) and infant mortality (SMR = 1.13; 95%CI 1.00–1.26). Our index highlights the relevance of considering the modes of living when devising deprivation indices or similar measures from Colombia or Latin America. This approach may provide different perspectives on the health-disease process and potential value for public health planning.

## 1. Introduction

Measures of deprivation, known generically as deprivation indices, are used to rank areas according to multiple indicators and may be used to monitor health inequalities, allocate healthcare resources, and study associations with the health status of individuals and populations [1,2]. Nevertheless, the theoretical underpinnings of most deprivation indices have been based on the context of Western Industrialized countries [3,4], with specific and very distinct social and historical contexts to those of the Global South. This also encompasses conceptualizing the health and disease processes and what determines them [5,6].

While conventional definitions of deprivation may be effective in certain contexts, they may fall short in explaining inequities and their health impacts in Colombia and other Latin American and Caribbean countries. Colombia is a multicultural and multiethnic upper-middle-income country located in the northeastern part of South America. Its geography is characterized by two oceans—the Atlantic Ocean to the north and the Pacific Ocean to the west—as well as the Amazon region and the Andean Mountain system. It consists of 32 administrative units known as departments, along with the capital district of Bogotá. With an estimated population of 52 million by 2024, Colombia ranks as the fourth-largest economy in Latin America. According to the 2018 census, approximately 4.9 million individuals identified as members of an ethnic community, including Afro-Colombians, Indigenous peoples, Raizales, and Palenqueros.

Colombia faced an important history of colonialism and has experienced a particular development process related to its complex geography [7], marked by armed conflict, internal displacement, and insufficient state policies [8]. Although it has seen a significant increase in economic indicators across the years, this has not been reflected in the reduction in social inequities, due among others to the persistence of historical conditions of inequity such as the concentration of land and socio-labor opportunities in urban territories and advantaged classes [9].

In this article, following the Latin American Social Determination of Health perspective, particularly the category Modes of Living supported by the construct of precarity, we aimed to develop an index of precarity in the modes of living at the department level in Colombia and assess its geographical distribution and usefulness in public health. We begin with an overview of the theoretical foundations for constructing the index, followed by the methodological deployment, results, and discussion.

### 1.1. The Latin American Social Determination of Health

Alternative theoretical explanations proposed by the social medicine and collective health movements in Latin America and the Caribbean have highlighted the need to reconceptualize health to transform the modes of action towards it [5,10,11]. Authors such as Almeida Filho and Breilh have proposed the social determination of health perspective as an alternative to some limitations of the more traditional perspectives (including, among others, a reductionist approach to health to risk factors) [12,13,14]. From this perspective, and following Breilh’s framework [6], the health of populations can be understood as the result of a multidimensional and dialectical (i.e., reciprocally constructed) social process between protective mechanisms that promote/generate health and destructive mechanisms that deteriorate it. Which varies for each human group, shaped by the interplay of processes at the singular (individuals and families), particular (social groups), and general (society as a whole) levels, and insofar as social class, ethnic, and gender relations are interwoven in it [6,15].

Understanding the collective patterns of material and symbolic social reproduction—which includes both observable and ideological elements that elucidate the persistence of social structures over time [16]—is fundamental. This starts with recognizing that social and collective processes cannot be reduced to the individual [17]. This awareness is vital, as health and disease arise from the social fabric of life, intricately linked to how societies are structured.

#### 1.1.1. The Modes of Living

The “modes of living” is a theoretical category developed to elucidate the relationship between structural social processes (such as social stratification) and micro-social processes (such as the styles of living). It designates the modes of existence and brings to the forefront the social dimension in which individuals are inscribed, accentuating its interdependent and historical nature.

The concept of modes of living originates in the foundational ideas of Marx, Engels, and Bourdieu, albeit initially under related terms like modes of production, social class, and habitus. Over time, it evolved to highlight “modes of existence” that reflect socio-economic hierarchies. Since the late 1980s, Latin American social medicine has further developed this concept, viewing the modes of living as a determinant instance of the health-disease processes and recognizing everyday life as the foundation for both material and symbolic social reproduction [18]. The category of modes of living has increasingly been approached in recent years for the study and interpretation of health inequities in the region [19,20,21,22].

According to Breilh’s framework, the modes of living can be understood as the patterns of existence through which various social groups sustain their social reproduction. These patterns reflect either heightened vulnerability to adverse conditions or an enhanced ability to capitalize on favorable processes [6]. Under this framework, the modes of living are organized into five dimensions: (a) Metabolic (ecosystem) relations, (b) Consumption, (c) Cultural-spiritual means, (d) Work, and (e) Organization and support. In contrast, the “styles of living” refer to the individualized expressions within a given mode of living [23]. A semantic difference between lifestyles and styles of living is proposed to be able to discern between the traditional approaches and the social determination of health [6].

In these interconnected dimensions, “metabolic (ecosystem) relations” describe how groups interact with their ecosystem, utilizing and appropriating natural resources for the reproduction of life and the creation of goods and means of production. The “consumption” dimension encompasses the social reproduction of daily life, addressing the group’s ability to access and utilize goods and services to meet basic needs. “Cultural-spiritual means” reflects the collective capacity to create and sustain values, perceptions, and practices that shape identity. The “work” dimension refers to how social groups produce their means of subsistence and establish conditions for productive activity. Lastly, the “organization and support” dimension involves the establishment of interdependent relationships within the group’s territory, including the capacity to mobilize collective resources for mutual benefit [6,24].

#### 1.1.2. Approaching Deprivation from the Social Determination of Health

Traditional deprivation measures are often based on the foundational work of Townsend and Carstairs [1,25], reflecting mainstream definitions of health and its determinants. These measures typically rely on a limited set of indicators to capture material conditions of disadvantage. However, this approach may overlook the complexity of the “modes of living” and the mechanisms generating inequity in other contexts, such as in Latin America. Conventional measures rely on aspects such as homeownership, car ownership, unemployment, or belonging to a low occupational class [1,3]. However, these criteria fail to capture other critical conditions that affect the living circumstances of population groups, particularly those less privileged. In Colombia, for example, not owning a home is common, with many families relying on rental agreements. Many additional aspects influence access to adequate housing that are not fully represented by simply lacking homeownership. While unemployment is undoubtedly significant, there are also hidden aspects that greatly impact the well-being of individuals and families. These include precarious working conditions and informal employment, which are particularly relevant in Latin America and the Caribbean [26]. Moreover, approaching social class through a traditional lens may not accurately reflect the complexities of class relationships within the country. Similarly, conventional measures may overlook context-specific material and symbolic dimensions of inequity. Such approaches may ultimately fail to fully understand the disparities present in a country with a deep and historical context of inequity. As a result, this narrow focus may not adequately address the broader structural and symbolic components that create and sustain inequities [27,28].

A comprehensive, epistemologically and contextually coherent definition of deprivation, as well as its operationalization through an area-level measure that considers the modes of living with the specificities of the local context, may provide a better picture of the configuration of inequities in the territories (e.g., by expanding the view on the range of determining mechanisms that create and reproduce inequities) and their influence on health and may provide additional insights for territorial and health planning. To fully capture these inequities, the concept of *precarity* offers valuable insight, addressing not only material deprivation but also the broader social vulnerabilities and power dynamics that shape modes of living in neoliberal contexts.

### 1.2. Precarity in the Modes of Living

Precarity is a broad concept that provides a historical contestation about capitalism and highlights the changes in class relations, social movements, and political struggles, particularly under the current neoliberal model [29]. It designates a form of governance in times of neoliberal domination, which is reproduced throughout the global order [30,31]. It emerged in scholarly work in the twenty-first century and has been explored by several authors in the last decades [30,31,32,33,34]. Two main viewpoints of precarity may be identified. On the one hand, it has been instrumental in academic discussions to explain the social mobilizations that have occurred in different parts of the world to denounce unemployment, the lack of stable jobs, affordable housing, and the dismantling of social welfare benefits that have accompanied the implementation of austerity policies of neoliberal regimes [29].

On the other hand, precarity serves as an overarching interpretive category for a context in which the forms of social reproduction are persistently fragile and unpredictable [30,31]. It encompasses not only job insecurity but also a broader mode of subjectivation marked by uncertainty and instability, limiting identity formation and solidarity [35,36,37]. Precarity intersects with precariousness [38], the latter being an ontological condition reflecting human interdependence and shared vulnerability, while the former is a politically induced state shaped by inequity and hierarchy. This condition results from social and political arrangements that restrict protective networks for certain groups, leading to increased instability and a more governable population [30,38]. Ultimately, it perpetuates fragilization, increasing exposure to disease, poverty, violence, and death [30,31,35]. This viewpoint also aligns, among others, with the term “Precariat”, which describes a social class facing insecure employment, incomplete contracts, and job instability, which hinders professional identity development. Individuals in the precariat also have limited civil, cultural, social, political, and economic rights. This instability forces them to adapt their life expectations, resulting in a loss of control over personal time and skills development [39].

While the concepts of precarious employment and precariousness have been introduced in public health research to mainly explore the association of employment, job, and housing conditions with health outcomes [40,41], they often approach a categorized notion of precarity that may leave aside the social arrangements and governing forms, which, in line with a more overarching viewpoint of precarity and the social determination of health perspective, we consider relevant for understanding the modes of living and to promote critical action towards inequity under the current neoliberal model.

Similarly, we believe that the concept of precarity has an encompassing notion incorporating deprivation. While the latter serves as a complementary tool through its empirical use, precarity might better conceptualize and explain the modes of living of different social groups and territories within a given social formation. It may more accurately reflect the historical characteristics of Colombia and other countries in the region. As such, referring to “precarity in the modes of living” allows us to point out that these modes of social reproduction do not occur randomly; on the contrary, they correspond to social patterns of differentiation, hierarchization, and exclusion that accumulate in certain social positions and groups [36,37]. Furthermore, the concept of precarity in the modes of living aids in comprehending the intersectional nature of the mechanisms that produce inequities. It underscores how membership in a social group influences their opportunities, the allocation of material resources, and shapes their self-perceptions, perpetuating a cycle of precarity across various dimensions. In this article, we, therefore, understand precarity in the modes of living as the expression of the harmful and destructive conditions driven by inequity in a social formation.

To our knowledge, no study has examined precarity in the modes of living at the ecological level in Colombia, nor has deprivation been approached through an alternative epistemological lens in this context. Existing research on modes of living in Latin America and the Caribbean has largely been theoretical [21,28] or based on qualitative approaches that may be challenging to operationalize within the current political and administrative frameworks. Additionally, few deprivation measures in Latin America approached the Latin American social medicine or the social determination of health perspectives, with only one study having referred to this literature directly [42]. Therefore, we sought to investigate how precarity in the modes of living is configured and distributed in Colombia and how it could potentially inform public health.

## 2. Materials and Methods

### 2.1. Research Aim

Develop an index of precarity in the modes of living at the departmental level in Colombia, assessing its geographical distribution and potential value for public health planning.

### 2.2. Study Setting and Data Sources

An ecological cross-sectional study with national administrative records from 2018 was conducted using the first administrative subdivision of the country as the unit of analysis, i.e., the 32 departments and the Capital District of Bogotá, with a heterogeneous population size ranging from around 51,000 to 7,968,000 inhabitants according to the 2018 population census.

Data for index development were obtained from two national population-based surveys developed by the Colombian National Statistical Office (Departamento Administrativo Nacional de Estadística (DANE)): the National Living Standards Measurement Survey (LSMS) (Encuesta Nacional de Calidad de Vida [43]) and the Large Integrated Household Survey (LIHS) (Gran Encuesta Integrada de Hogares [44]), both from 2018. Both were representative at the national and department levels. Further information about the surveys may be found in the supplementary material (Supplementary Material, Section S1, Text S1).

The LSMS surveyed 89,522 households and 283,012 individuals, comprising the final analytical sample from this survey. While the LIHS surveyed 250,856 households and 826,533 individuals, the final sample used for constructing indicators was 376,676 individuals corresponding to those occupied at the moment of the survey.

Data on live births and child deaths were used to analyze the usefulness of the index and were retrieved from the national vital registries for the years 2017 to 2019, with a total of 26,290 under five years deaths, 21,664 corresponding to infant deaths, and 1,948,479 live births in the country within that period.

### 2.3. Index Development

#### 2.3.1. Index Dimensions

We used Breilh’s five dimensions of the modes of living: Work, Consumption, Cultural-spiritual means, Organization and supports, and Metabolic (ecosystemic) relations, and an additional dimension to reflect its interrelated nature with power relations as explained in Breilh’s framework [6].

#### 2.3.2. Selection of Indicators

Indicators that were representative of the five dimensions of the modes of living, as well as of power relations, were selected for analysis based on a literature review of the modes of living [6,18,22,24,28,45,46] as well as of other deprivation indices developed worldwide [1,2,3,4,47,48,49], with a particular focus on those developed in Latin America and the Caribbean [42,50,51,52,53,54,55]. The indicators were validated through discussions among the researchers and expert advice, particularly for those without a consistent pattern in the literature.

Most indicators were included from the LSMS, while several related to the work dimension and a social class classification based on the same theoretical perspective were retrieved from the variables available in the LIHS. Indicators were calculated from the available variables in the surveys by using the appropriate numerator and denominator (Tables S1 and S2 Supplementary Material). Depending on the indicator, the proportion of the population with a given characteristic or the median value for a given characteristic per department was calculated to construct each indicator. Indicators were created so that higher proportions represented higher precarity. The difference between rural and urban composition was considered when building some indicators. Only indicators representing a complete or a partial state of precarity were included in analyses. Thirty-nine indicators were initially selected and introduced into the analysis (Table S1 Supplementary Material). Some indicators were further categorized by sex to explore a possible gender divide in sensitivity analyses (Table S2 Supplementary Material).

#### 2.3.3. Sequential Principal Component Analysis

Principal Component Analysis (PCA) was chosen as the technique for index development as it allows a data-driven approach and avoids selecting indicators based exclusively on expert choice. Sequential PCAs were carried out following the procedures of Duque et al. [2] and Lalloué et al. [48], as well as the R functions developed by Lalloué et al. within the package SesIndexCreatoR for the development of socioeconomic indices in the R environment [56], with some modifications. Indicators with high skewness (>1.5) were square-root transformed prior to the sequential PCA, as it has been suggested that variables with high skewness may outweigh the results [57]. Before starting the sequential PCA, variables were standardized to have a mean of 0 and a standard deviation of 1. Further explanation of the sequential PCA may be found in the supplementary documents (Text S1, Section S1, Supplementary Material). The indicators constitutive of the final PCA were further discussed within the team to explore theoretical validity. An additional PCA was performed, incorporating indicators considered relevant for the precarity construct, which could have been excluded in the last step for statistical reasons.

We extracted index scores for each department and standardized the index to have a mean of 0 and a standard deviation of 1. Departments were then further classified into quintiles of precarity, with the first and fifth quintiles representing the least and the most deprived, respectively, and were depicted using choropleth maps.

Internal consistency of the theoretical construct was assessed through Spearman correlation tests of each indicator with the final index and assessing the performance of the McDonald Omega coefficient. External consistency was assessed through Spearman correlation tests with the Multidimensional Poverty Index (MPI) developed by the National Statistical Office [58], as it is one of the standard measures for territorial planning in the country and has been useful in analyzing territorial health inequalities in the last years [59].

### 2.4. Spatial Autocorrelation Analysis

Global and local spatial autocorrelation analyses were conducted to assess the spatial distribution of the index. Global Moran’s I and Local Indicators of Spatial Association (LISA) were conducted to test for this association. Briefly, the Global Moran’s I is a test for overall spatial autocorrelation, with significant results ranging from −1 to 1 indicating a spatial process and a value of 0 indicating a random distribution. In parallel, LISA allows for a more detailed explanation of the spatial process at the local level by classifying the observations into five categories according to their neighbors’ values [60].

Due to the polygon structure of the data, its size, and the presence of the islands of San Andrés and Providencia, a k-nearest neighbor (k = 4) matrix was developed for all the analyses. Sensitivity analyses were conducted with Adaptive Kernel, Rook, and Queen contiguity matrices. For the last two, the islands were excluded from the analysis. To increase the robustness of Moran’s I, Monte Carlo simulations with 9999 permutations were performed. Interesting locations were considered with a pseudo-*p*-value of 0.05.

### 2.5. Regression with Health Indicator

Under-five mortality rate (U5MR) and Infant Mortality rate (IMR) were considered good indicators to look at the usefulness of our index for public health purposes due to its reported association with socioeconomic and environmental conditions in the literature [59,61]. Under-five mortality rate was defined as the total number of deaths in children aged 0 to 59 months divided by the total number of live births during the same period and multiplied by a constant of 1000. Infant mortality was similarly defined using the total number of deaths in children aged 0 to 12 months as the numerator.

Spatial and non-spatial models were developed to assess the association of precarity at the department level with U5MR and IMR. Negative binomial regression models were conducted with the number of deaths as the dependent variable and the precarity index as the only predictor, using the number of live births as an offset term. Standardized mortality ratios (SMR) were obtained as a result. Sensitivity analyses were conducted with a spatial cross-regressive model (Spatially lagged X model) with the adjusted rates. To increase the robustness of the findings, all regressions were performed by pooling live births and deaths data from 2017 to 2019. All analyses were conducted using R v.4.0.1 and STATA v.14 (StataCorp, College Station, TX, USA).

## 3. Results

### 3.1. Index of Precarity in the Modes of Living

Our index included 20 indicators based on the first component retrieved from the final PCA, which accounted for 70.8% of the total variance of the data. It kept indicators from four dimensions of the modes of living—metabolic relations, consumption, culture and spiritual means, and work—as well as power relations with a differential contribution of each indicator to the main index (Table 1).

The dimension “organization and supports” did not remain as its representative indicators did not contribute meaningfully to the iterative PCA. Additionally, although in the final PCA, the indicator “Population older than 65 years” was not retrieved initially, we decided to keep this indicator as part of the final index as it was considered to provide a more comprehensive representation of precarity across the age spectrum in the country [62].

**Figure 1:**
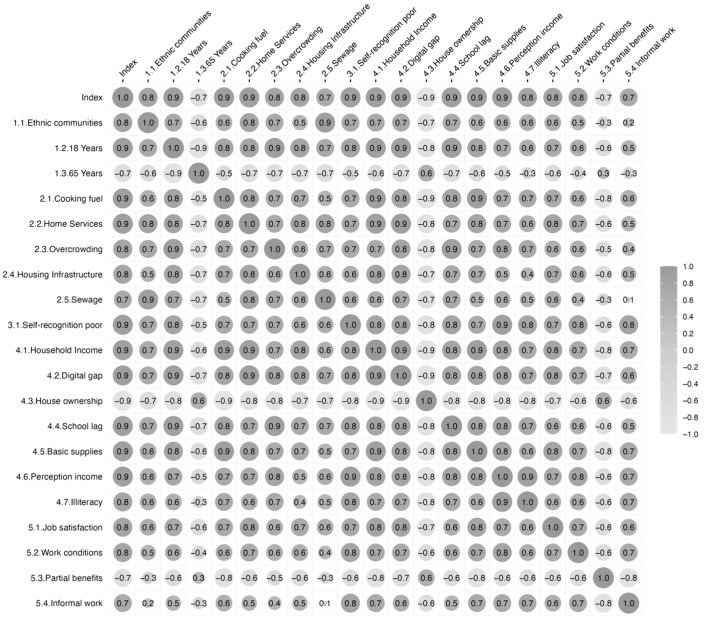
Correlation matrix between indicators constitutive of the index and the index of precarity. Results from Spearman correlation. Darker circle shows positive correlation, lighter circle shows negative correlation. Numbers in indicator’s labels refer to the order in Table 1.

The first component of the PCA was positively correlated with indicators representing higher precarity, such as insufficient household infrastructure, and negatively correlated with indicators of partial precarity, such as partial employment benefits (Figure 1). The latter type allows contrasting those departments with less precarity. The indicators showed a considerable variation across study areas, ranging from 1% to more than 90% depending on the indicator (Table 1 and Supplementary Figures S1–S5), and all exhibited a high correlation with the index (Figure 1).

**Figure 2:**
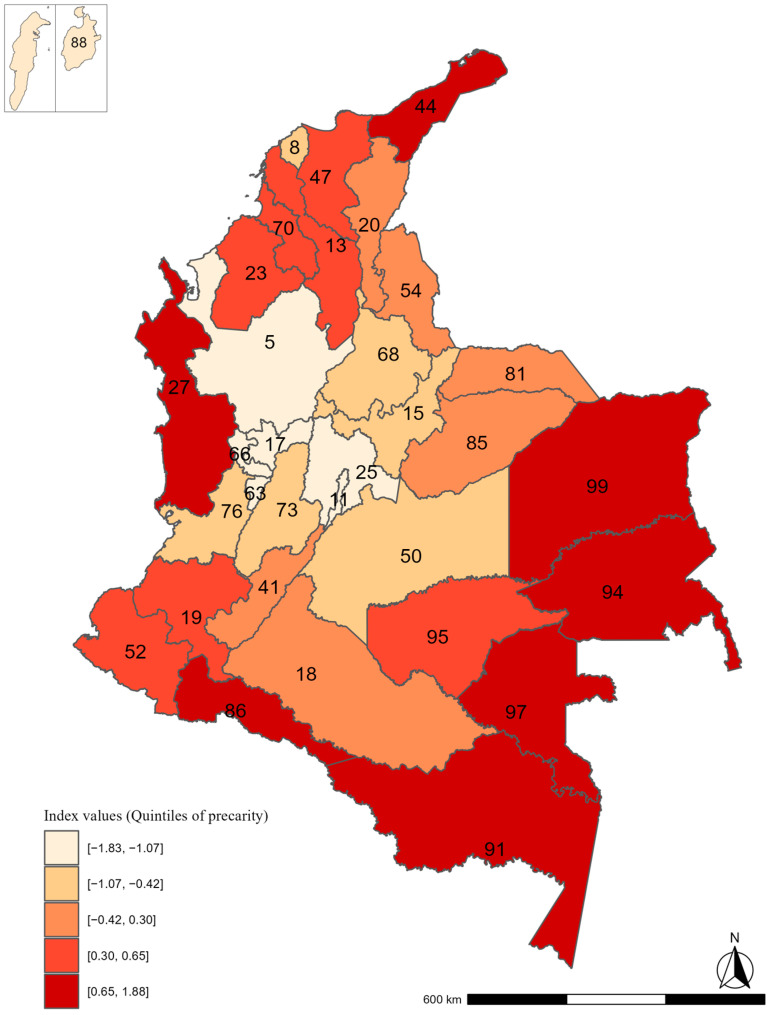
Geographical distribution of the precarity index at the department level in Colombia. Higher values (darker) represent higher deprivation. The Islands of San Andrés and Providencia (88) were enlarged from their original size for visualization purposes and do not follow the depicted scale and location. For reference, Bogotá (the capital city) is labeled with the number 11.

Our index had a good internal consistency with a 0.98 omega coefficient and exhibited a strong correlation with the Multidimensional Poverty Index (MPI) (0.92). When looking at its geographical distribution, a visual pattern of higher precarity in the periphery and lower precarity towards the center of the country was observed (Figure 2). No gender divide was perceived when performing sensitivity analyses differentiating some indicators by sex, as the same final indicators as those in the overall analyses were selected. Nevertheless, for some indicators, the mean proportion was slightly higher for women (i.e., 4.1 and 4.6 in Table 1) and for men (i.e., 5.2 in Table 1) (Table S2, Section S1, Supplementary Material).

### 3.2. Spatial Autocorrelation of Precarity

**Figure 3:**
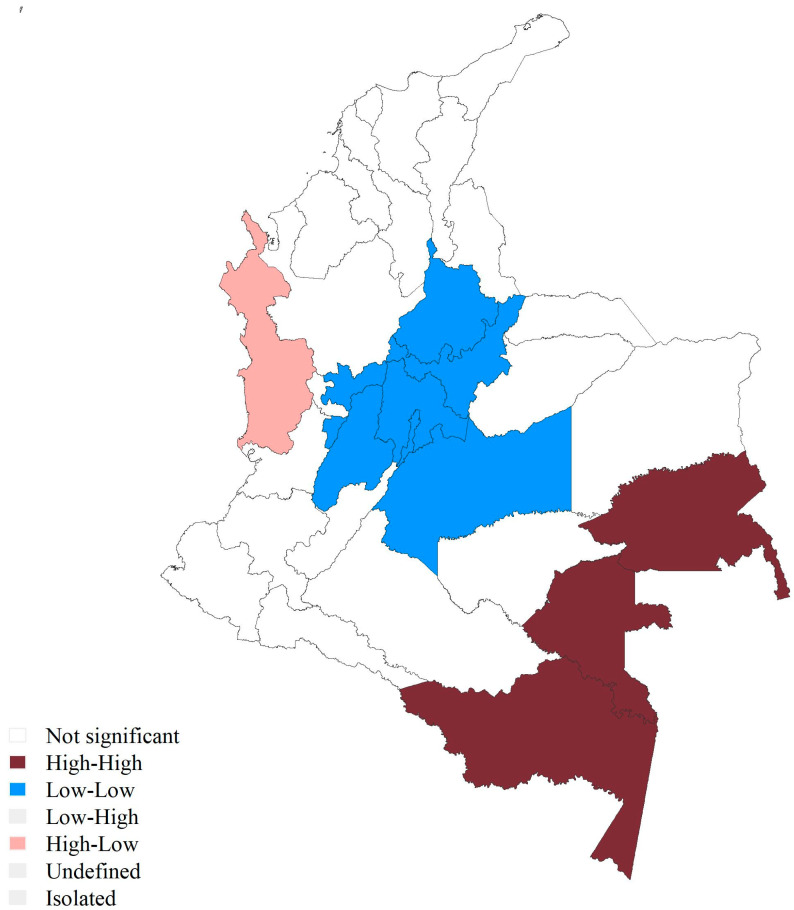
LISA clusters of the precarity index. High-High corresponds to a cluster of high values surrounded by high values. Low-Low corresponds to a cluster of low values surrounded by low values. High-Low corresponds to a high value surrounded by low values. White corresponds to non-significant values (non-significant). The Islands of San Andrés and Providencia were classified as non-significant.

Global Moran’s I indicated a significant positive spatial autocorrelation of the precarity index across departments (I = 0.34, *p* < 0.001). This was robust to the use of different spatial weight matrices and permutations. When looking at the local indicators of spatial association, a significant cluster of high values surrounded by high values (high-high) was found in the southeastern region of the country, including the departments of Amazonas (91), Vaupés (97), and Guainía (94). In parallel, a significant cluster of low values surrounded by low values (low-low) was found towards the center in the Andean region, constituted by the departments of Risaralda (66), Quindío (63), Caldas (17), Tolima (73), Meta (50), Cundinamarca (25), Boyacá (15), Santander (68), and Bogotá (11) (Figure 3). There was an outlier of a high value surrounded by low values (high-low) corresponding to the department of Chocó (27) in the Pacific region. Similar patterns were found in sensitivity analyses (Supplementary Figure S6).

### 3.3. Association with Public Health Indicators

We found an association of our precarity index with under-five mortality at the department level, with an increased mortality ratio for each standard deviation increase in the index (SMR = 1.19; 95%CI 1.08–1.31). A similar association was also found for the index when conducting the spatial lag X model, but the spatial lag was not significant (Table S3 Supplementary Material). Similar results were found when looking only at infant mortality (SMR = 1.13; 95%CI 1.00–1.26) (Tables S3 and S4 Supplementary Material).

**Figure 4:**
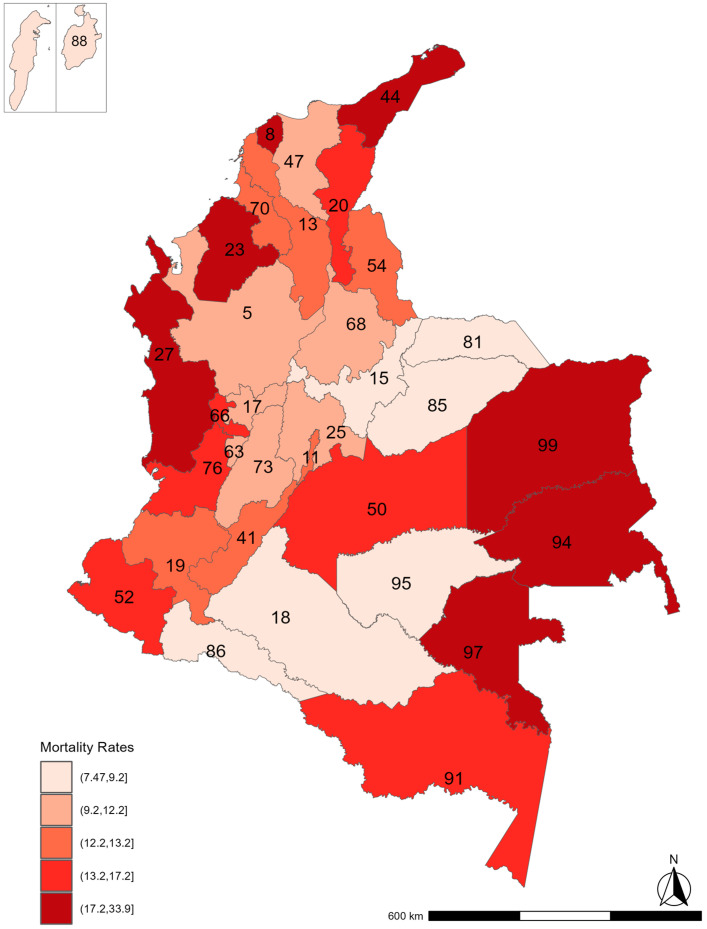
Geographical distribution of under-five mortality rates by quintiles in Colombia between 2017 and 2019. The Islands of San Andrés and Providencia (88) were enlarged from their original size for visualization purposes and do not follow the depicted scale and location. For reference, Bogotá (the capital city) is labeled with the number 11.

When looking at its geographical distribution, a similar pattern of higher rates was found in the periphery of the country, corresponding to the departments with higher precarity. However, the distribution was dissimilar for the rest of the quintiles (Figure 4).

## 4. Discussion

In this cross-sectional ecological study, we developed an index of precarity based on the theoretical category of the modes of living from the Latin American Social Determination of Health perspective. Our index showed a good overall performance and represents the dimensions of metabolic relations, consumption, cultural-spiritual means, work, and power relations. It does not follow a random distribution in the country, with a visible pattern of higher precarity towards the periphery and lower precarity towards the center. We also found an association of our index with child mortality at the ecological level, with an increased mortality ratio with each increase in the index values.

### 4.1. Index Constitution

#### 4.1.1. Metabolic (Ecosystem) Relations

The indicators representing the metabolic relations dimension reflect much of the material deprivations that individuals and communities face, particularly those related to housing. Adequate housing encompasses a set of several conditions and has a deteriorating or protective role for health [63]. The indicators kept are shared by other conventional measures in Colombia and elsewhere, such as the Multidimensional Poverty Index (MPI) and the Index of Unsatisfied Basic Needs (UBN), and reinforce the need to address structural problems of housing and its relationship with the environment in the territories. Contrary to what is found in the more conventional indices where only walls and flooring material are considered, our index underscores the need to consider housing infrastructure as a whole by including ceiling materials in its construction. Similar proposals are found in Argentina [52] and Ecuador [42]. This is particularly important when global warming is exacerbating and where the lack of any constitutive structures may put families and communities in a vulnerable situation.

Another important aspect of this dimension is the consideration of access to basic services. Although conventional indices include only access to water as the one that might provide a better consideration of the deprivation that people face due to the characteristics of communities in Colombia and to the interrelated dynamics of the different dimensions of the modes of living, we considered a more comprehensive idea of access to services, also including the lack of access to energy and garbage collection. This is consistent with the incorporation of such indicators in measures developed in countries with similar characteristics, such as Mexico [54] and Brazil [55], and calls attention to the need to consider how the lack of access to different basic services could limit communities’ and individuals’ ability to develop their daily living fully and without constraints.

#### 4.1.2. Consumption

An important number of indicators represented the consumption dimension; some are shared with other measures, such as school lag and illiteracy found in the MPI, as well as the lack of house ownership found in traditional deprivation indices [1]. Nevertheless, our index includes additional indicators not considered in conventional measures. First, the construct of the digital divide, one of the indicators with the highest contributions to the index, reflects the outstanding deficit of the country in this matter and puts to the front the difficulty that people may face when trying to cope with the fast nature of digital development, notably when lacking the material tools for confronting the “information age” [64] and the new economies and job arrangements based on it [65,66,67,68]. Second, the idea of a lack of material goods, based on the lack of basic home supplies, draws attention to the limitations that some individuals and families may have to supply essential daily activities such as cooking, adequately preserving food, and doing their laundry (a relevant indicator particularly associated with an improvement in the living conditions and opportunities for women under the historical structure of care in the country). Finally, indicators related to family income reflect the need to consider the ability to afford basic expenses, as given by a median family income below the minimum wage level. Additionally, our index not only included indicators from a more objective dimension but also considered a subjective dimension of consumption through the perception of having an insufficient household income for the basic living expenses, consequently enabling the social reproduction of the family, highlighting the importance of accounting for the subjective experiences perceived by people when developing measures intended for planning purposes.

#### 4.1.3. Cultural-Spiritual Means

Along the same lines, the indicator representative of the dimension cultural-spiritual means underscores how people’s perception of being poor relates to their social and material conditions. Those with higher precarity could experience a higher self-recognition of being in distress, which could, in fact, affect their health and prevent them from integrating and accessing services such as healthcare. People who perceive themselves as poor may embody this status, affecting all other dimensions of life and remaining in a negative cycle that could further exacerbate health inequities [69].

Peralta et al. reported a similar indicator as constitutive of their index in Ecuador [42], which calls attention to considering the subjective perception of precarity as experienced by individuals and communities for more comprehensive and community-informed planning. How individuals perceive themselves should be an important consideration in the analysis of inequalities, since these perceptions imply a prior and historical conception that is created in the territory and in the relationships with others. This refers to material and symbolic aspects that impact the meanings and interpretations of what can be thought and done and what can be socially reproduced as correct, normal, and acceptable [70]. The self-perception of poverty goes through symbolic and interpretive or culturally reproduced processes. This does not occur only from the actions and thoughts of those who perceive themselves as poor but also from how others perceive them. The institutions that reproduce discourses and visions about poverty could reinforce patterns of understanding and social labels to the detriment of individuals, families, and territories living in poverty [71].

It’s important to note that the culture-spiritual means dimension should not be reduced to the perception of a single individual. Instead, it should encompass the historical and intergenerational aspects associated with it. This consideration is especially relevant when acknowledging that there may be intergenerational transmission of knowledge and cultural identities [72]. Thus, our index can only offer a partial view of this dimension, and further studies that account for this transmission may be necessary. Further research with additional indicators, such as those proposed within some models approaching culture and cultural capital [73,74] may provide a more comprehensive assessment of this dimension. Nonetheless, the conceptualization and measurement of “culture” are complex [75] and might not be fully achieved by quantitative methods, requiring exploration based on qualitative approaches that allow a better understanding of the full extent of complex social dynamics [76]. Additionally, the cultural-spiritual means of individuals and populations are intricately connected to other dimensions of the modes of living and to the power dynamics of societies, which entails that other indicators from our index also encompass this dimension, clear examples being belonging to an ethnic community or a specific age group.

#### 4.1.4. Work

The work dimension, interestingly, was represented by indicators that have not been referred to in other measures, including the consideration of having inadequate conditions at the job place and the perception of low job satisfaction. This is particularly important from a health perspective, as some studies have shown a relationship between job satisfaction with mental health and subjective physical health [77], although those studies have been conducted at the individual level. People experiencing precarious conditions at work may fall into a negative cycle of social deprivation that could affect their health and well-being [78], and that could translate into a similar shared experience of communities in different territories. Conversely, some studies have also shown that workers in precarious conditions might develop a sense of collective power when they can organize and move towards a common goal, counteracting some of the negative consequences of these conditions [79].

Moreover, although the informal work/lack of complete social security is a recognizable feature of Latin American societies [80] and is prominent in Colombia [81], this indicator had a smaller contribution to the index when compared to the rest; nevertheless, it was kept by the data-driven approach within the work dimension over other indicators that have been traditionally used in similar measures (such as unemployment) and reflects the importance of considering the informal sector for policy planning within the country. It is important to consider that several indicators from the work dimension were constrained to the employed population, so social segments that are not represented might have been excluded from the analysis. Therefore, the inequity gaps could have been greater than those reflected in our index. Further research exploring the precarity in the work dimension across all social segments and the subjective experiences on the configuration of precarity is warranted.

#### 4.1.5. Organization and Support

When conducting the analyses, the dimension organization and support were excluded from the final index. This dimension might not have been represented at its fullest by the data available in the surveys and by the indicators incorporated into the analysis, which included the use of government subsidies, the feeling of insecurity, and being a victim of a criminal act (Supplementary Table S1). A similar result was reported by Peralta et al. when looking at their dimension of social environment/social capital in Ecuador [42]. Nonetheless, other indicators might better reflect precarity within this dimension, such as those proposed within social cohesion indices, including, among others, social bonds, interpersonal trust, and respect for diversity [82], or other indicators that better represent the experience of solidarity that were not found in the national data sources explored.

#### 4.1.6. Power Relations

Our index also exposes power dynamics based on ethnicity and the life course, with children and ethnic communities being considered part of the precarity construct. The inclusion of indicators related to the ethnic composition of communities has been reported in other indices in the region [42,55]. It highlights the relevance of considering the historical and territorial discrimination and segregation that these communities have faced and its interrelatedness with other conditions of material and social deprivation [83,84]. Similarly, children are vulnerable to their living conditions and might experience more predominantly the effects of deprivation [85]. Although the indicator related to older adults was not included by the data-driven approach, we kept it as it was considered important to better reflect precarity in the country [62] by providing a broader picture of the circumstances and power relations in two vulnerable periods of the life-course. While an indicator of social class was initially included for analysis (Supplementary Table S1), it was not kept in the iterative process, possibly because a high proportion of the population within all departments was classified in the less privileged social classes and did not allow contrasting the territories. Consequently, we hypothesize that our index might indeed be reflecting the precarious conditions of the less privileged social classes in the country, and we considered it redundant to keep it as an indicator at this level. Further analysis at finer scales might provide a different picture, where the social class dynamics can be better revealed.

Although including the proportion of ethnic communities or other social groups in an index may be seen as masking the precarity experienced by those groups, due to the historical characteristics of the ethnic communities in Colombia, we consider it relevant to incorporate this indicator to be able to capture, at least partially, the systematized state of oblivion, general discrimination, and influence of conflict experienced and embodied by them. Recognizing that it does not correspond to an intrinsic trait but rather constitutes a social condition produced by the institutional arrangement. This is particularly relevant with the number of people belonging to ethnic communities in the country. While ethnic communities might be proportionally higher in some territories, they are distributed heterogeneously across the entire country [86]. Accordingly, what our indicator may be showing is rather the convergence of destructive processes in some territories and not only the predominance of those communities in specific territories.

### 4.2. Distribution of Precarity and Potential Value for Public Health Planning

Our index shows a clear pattern of precarity in the periphery of the country, involving territories on the Atlantic and Pacific coasts and all the departments of the Amazon region, and less precarity in the center towards the Andean region. This is consistent with the findings of other studies that identify a pattern of unequal socioeconomic development between the central (Andean) region, the main capital cities, and the rest of the country [87,88]. This might be the result of a combination of historical processes, both structural and subjective, in which political and economic power was concentrated in the Andean region, characterized by higher levels of urbanization, institutional development, and technical-productive capacities, as opposed to the peripheries of the country, with a more widely distributed rurality, lower population densities, lower institutional development, the concentration of ethnic populations in some territories, and a greater impact of the armed conflict [9,89].

Recent studies that analyzed the spatial distribution of the multidimensional poverty index and the unsatisfied basic needs index showed similar results to those found in our study. The departments of Chocó in the Pacific and those in the Caribbean and southern regions exhibited higher levels of poverty and unmet needs, while the central regions of the country demonstrated better conditions [90,91]. The data used in those studies were derived from the general population census. In contrast, our findings are based on data from population surveys because the census did not adequately capture the various dimensions of the modes of living. Although the overall distribution of precarity may align across different indices and data sources, it is crucial to emphasize the narratives behind each indicator. Planning exercises should consider dimensions that are often overlooked, as they could lead to a richer understanding of precarity. Our index provides an alternative way to explore these narratives and may encourage further research that delves into the mechanisms producing inequities. Moreover, future research should investigate innovative methods for operationalizing the modes of living by combining survey and census data and triangulating them with qualitative information. This approach could inform administrative efforts to collect additional data, ultimately providing a more comprehensive picture of precarity in the country.

We also found a positive association of our index with under-five and infant mortality, indicating that departments with higher precarity show higher mortality. Similar associations have been found at the municipal level with the Multidimensional Poverty Index (MPI) [59] and with indicators of socioeconomic and environmental conditions at the department level [92], which might support the usefulness of our index for health planning and research at this level.

Our index highlights the importance of considering the modes of living as a central category in the process of determination of health and wellness. Our results underscore the need to consider the interrelated nature of the different dimensions for a comprehensive understanding of the precarity that social groups and their territories face. Along these lines and as reflected in the index constitution, precarity is characterized by situations where instability, insecurity, temporality, and deterioration of working conditions and wages converge and multiply, entailing a radical instability of living conditions, which causes a substantial limitation for the formation of identities and the deployment of solidarity. A process that does not occur randomly but corresponds to social patterns of differentiation, hierarchy, and exclusion, which accumulate in certain positions and groups [36,37].

Still, while the concept of precarity is instrumental for conceptualizing and explaining the inequity in the modes of living, caution should be taken in using a general theory that may replicate or be absorbed in state categories of the poor and vulnerable that promote territorial stigmatization [35].

The notion of precarity, from an overarching viewpoint, is proposed to reposition the demands of a progressive political action whose horizon is the radical defense of the right to life. This is grounded on the understanding that all life involves an intrinsic existential condition of vulnerability, interdependence, and harmfulness (precariousness). It underlies the ethical and social obligation to provide and promote egalitarian basic social supports and healthy and sustainable modes of living (e.g., food, shelter, work, health care, education, the right to mobility and expression, protection against oppression, etc.) that minimize such precariousness and its unequal distribution. In turn, such action should contend with those situations where the necessary political and social conditions of protection do not exist, which exacerbate violence and deepen inequity, preventing the development of a life worth living (precarity) [30,31,35]. Also aligned with the concept of the Precariat, understanding the complexities of precarity in the modes of living is essential for characterizing the current social context in Colombia, as well as in Latin America and the Caribbean. This is particularly important when considering the features of their labor markets and how these intersect with other processes that contribute to social precarization.

To build healthy and sustainable modes of living, it is therefore imperative to stand from a perspective where shared precariousness and the existence of induced political conditions of precarity are recognized. This could drive a greater social demand for the universalization of all rights that allow every person to prosper and live a healthy and fulfilling life. Additionally, it could drive the broadening of social coalitions that challenge the expansion of institutionalized violence brought by the dismantlement of welfare policies and other forms of production, exploitation, and distribution of precarity under a neoliberal model.

### 4.3. Strengths and Limitations

Strengths of our study include using representative records at the national and department level, analyzing the spatial distribution with statistical criteria and not only through thematic maps, and developing an index following a mixed theory and data-driven approach. Our index is also one of the few attempts to consider area-level precarity from alternative theoretical perspectives in the Latin American and the Caribbean region. It highlights the importance of considering the different constitutive dimensions of life and its social reproduction as an interrelated continuum and brings to the table the need for assessing indicators that have not been considered in conventional analyses.

Our study also has some limitations. First, the scale at which analyses were developed may hide internal dynamics of precarity within the departments and cities, particularly considering the high internal heterogeneity of the territories. This was related to the availability of data from National Surveys where several dimensions of the modes of living could be operationalized. Further work at finer scales would be required, particularly for developing association studies with health at the individual level. Second, while the selection of indicators was detailed, it was not exhaustive of all the available data sources in the country. Along these lines, although excluding the dimension of organization and support from the index might miss a complete picture of the modes of living, the available indicators in the data sources used might not be the best representation of this dimension, as might be the case in other exercises and data sources (i.e., at the local level). We, therefore, did not consider it appropriate to select one of the indicators available or to retain them all to keep this dimension in the final index. Additional analyses that consider other information about the dimensions of organization and support and cultural-spiritual means, as well as other characteristic features of inequity in Colombia, such as conflict, internal displacement, and economic and land use of the territories, could improve the understanding of precarity in its regions.

Additionally, we were unable to explore several dimensions that may inform the intersectional nature of inequities, such as the experiences of LGBTQI+ communities or people with disabilities, due to a lack of data or insufficient representation among informants. Moreover, some population groups may not have been included in official surveys, which can lead to an incomplete understanding of the patterns of precarity and the perceptions surrounding them. Still, our study is informative about some of the power relations that occur in the country, and it is relevant for informing future research. Further studies that explicitly incorporate an intersectional analysis framework and aim to analyze data from other population groups not covered in our research could provide valuable insights into how power relations impact the modes of living of different communities. Similarly, understanding the construction of identities and their intersectionality, as well as how these may be reproduced intergenerationally in culture and contribute to social inequality, is often difficult to capture using quantitative approaches. Qualitative methods may better capture these complexities and are essential for gaining a deeper understanding of these dimensions.

Third, although our index is based on the theoretical proposal from the social determination of health, some limitations may arise with our fundamentally survey-based quantitative approach and the lack of another type of information that could provide a broader picture of the territorial realities and the processes of subsumption/embodiment of precarity of communities and individuals [17]. Nevertheless, our study provides an exploration of the modes of living at the policy-administrative level. It may open possibilities for developing further research in the field by deepening the qualitative, historical, and dialectical frame of the precarity of the territories and social groups in Colombia. Future studies on the precarity in the modes of living would benefit from qualitative approaches that effectively capture the dialectical relationship between processes of precarization—driven by specific social arrangements—and the agency and resistance of individuals and communities, which ultimately shape the distinctive characteristics of the modes of living among social groups. In this context, mixed-method approaches that integrate qualitative data and direct engagement with communities offer an alternative to more comprehensively operationalizing Breilh’s framework, as they may provide a more nuanced understanding of the lived experiences of precarity, something that surveys alone can only superficially capture.

Finally, while the importance of considering measures that account for gender differences has been highlighted in the literature [93], we did not find differences in the constitutive indicators of our index when performing sensitivity analysis differentiating indicators of the work, consumption, and culture and spiritual means dimensions by sex. This might be related to the scale being analyzed as well as to the selection of less sensitive indicators from a gender perspective. Considering the gender divide is still imperative as it reflects the power relations that occur in societies and particularly in countries from a region without an adequate care infrastructure or political agenda [94], where women carry the burden of caring for children and older adults, in addition to other unpaid activities that could exacerbate social and health inequities [95]. Further work at finer scales and analyzing indicators that can more precisely reflect the gender differences in Colombia is warranted.

## 5. Conclusions

Addressing health inequities requires a broader perspective on health and disease, incorporating an understanding of the material and symbolic conditions of the existence of social groups and the ways they interrelate and impact health. Our index of precarity in the modes of living offers an alternative approach for both analytical and planning purposes, encouraging policymakers to adopt a more comprehensive view of the conditions that determine health and the indicators that inform their decision-making. In Colombia, it is essential to address the forces perpetuating precarious modes of living to transform the enduring patterns of precarity experienced by the territories and their communities.

## Figures and Tables

**Table 1:** Indicators constitutive of the index of precarity in the modes of living in Colombia, 2018.

Theoretical Dimension	Indicator	Minimum *	Median *	Maximum *	Contribution to Index (%)
1. Power relations	1.1. Ethnic communities (Afro-Colombian, Indigenous, and Rom communities)	0.45	10.5	95.5	4.0
	1.2. Population younger than 18 years	23.01	33.2	49.7	6.1
	1.3. Population older than 65 years	3.13	7.4	11.8	3.6
2. Metabolic (ecosystem) relations	2.1. Cooking with potentially dangerous sources (solid fuels)	0.08	15.9	81.5	5.6
	2.2. Lack of access to complete home services ^a^	0.92	32.2	83.1	5.6
	2.3. Overcrowding	3.4	13.9	39.9	5.0
	2.4. Insufficient housing infrastructure ^b^	6.2	41.1	99.8	4.4
	2.5. Lack of adequate sewage	0.39	13.4	71.6	3.8
3. Cultural-spiritual means	3.1. Self-recognition as poor	14.7	45.3	78.4	5.5
4. Consumption	4.1. Household income lower than the national minimum wage ^c^	8.97	35.3	74.4	6.3
	4.2. Digital divide ^d^	24.1	61.2	99.01	6.3
	4.3. Lack of house ownership	4.7	27.3	43.2	6.0
	4.4. School lag (backwardness) ^e^	5.8	13.1	26.7	5.8
	4.5. Lack of basic supplies ^f^	13.3	52.9	92.1	5.9
	4.6. Perception of insufficient household income for basic expenses	22.2	42.4	68.5	5.4
	4.7. Illiteracy ^e^	1.5	11.4	30.6	4.3
5. Work	5.1. Low job satisfaction	17.1	28.8	50.6	4.7
	5.2. Inadequate conditions at work ^g^	25.4	57.6	80.2	4.3
	5.3. Partial employee benefits and perks ^h^	1.02	11.05	48.5	3.8
	5.4. Lack of social security/Informal work ^i^	48.2	79.2	89.02	3.4

* Values indicating the percentage of the population: ^a^. Defined as lacking at least one of the following: water, electricity, or garbage collection. ^b^. Defined as lacking at least one of the following: adequate floors, ceilings, or walls. Traditional indigenous housing was excluded from this indicator, as those need to have special considerations on a specific case basis. ^c^. The minimum wage for the year 2018 was 781.242 pesos (roughly equivalent to USD 262 according to a 2.984 Colombian pesos conversion rate). ^d^. Defined as households without an Internet connection or with an Internet connection but without a computer/mobile phone. ^e^. Calculated for the household (at least one member of the household with illiteracy and for school lag at least one school-age child). ^f^. Defined as lacking at least one of the following: fridge, stove, or washing machine. ^g^. Defined as reporting at least one of the following: inadequate physical conditions, inadequate psychological conditions, or work overload. ^h^. Defined as lacking at least one of the following: paid vacation leave, Christmas bonus, and severance package. ^i^. Defined as lacking pension scheme and occupational risk insurance.

## Data Availability

Data for the study were accessed from public databases from the National Statistical Office of Colombia (DANE) available at (https://www.dane.gov.co/) (accessed on 5 March 2021).

## Supplementary Materials

The following supporting information can be downloaded at: https://www.mdpi.com/article/10.3390/ijerph22040537/s1, Table S1: Initial set of indicators introduced into the analysis; Table S2: Indicators constitutive of the final index of precarity in the modes of living and its operationalization; Figure S1: Geographical representation of indicators of power relations; Figure S2: Geographical representation of indicators from the metabolic (ecosystem) relations dimension; Figure S3: Geographical representation of indicators from the culture and spiritual means dimension; Figure S4: Geographical representation of indicators from the consumption dimension; Figure S5: Geographical representation of indicators from the work dimension; Figure S6: Results of the Local Indicators of Spatial Association (LISA) on the index of precarity using different spatial weights matrices; Table S3: Results from a negative binomial regression model assessing the association of the precarity index and under five and infant deaths; Table S4: Results from spatial lag x regression models assessing the association of the precarity index and under five and infant mortality rates. References [6,43,44,48] are cited in the supplementary documents.
