# Supplementary material for: Precarity in the Modes of Living: Proposing an Index for Studying Health Inequities at the Ecological Level in Colombia
Source: Int J Environ Res Public Health. 2025 Apr 1;22(4):537. doi: 10.3390/ijerph22040537 (PMC12026722; doi:10.3390/ijerph22040537)
Supplement: Supplementary file 1 [file ijerph-22-00537-s001.zip › ijerph-3325018-supplementary.pdf]

Supplementary material. Precarity in the Modes of Living: proposing an index for studying health inequities at the ecological level in Colombia

Hugo-Alejandro Santa-Ramírez, Andrés-Felipe Ramírez-Giraldo, Hugo Pilkington, Carme Borrell, and Gabriel-Jaime Otálvaro-Castro on behalf of the group *Inequidades, clase social y salud: una cartografía desde los modos de vida en Colombia*.

Table of contents

- Section 1. Index of precarity and constitutive indicators 2 - 9
- Section 2. Spatial Autocorrelation Analysis 10
- Section 3. Regression Models with Health Indicators 11

Section 1. Index of precarity and constitutive indicators

Text S1. Data and index development

Data Sources

The National Living standards measurement survey (LSMS) (Encuesta Nacional de Calidad de Vida) and the Large integrated household survey (LIHS) (Gran Encuesta Integrada de Hogares) are developed by the Colombian National Statistical Office (Departamento Administrativo Nacional de Estadística (DANE)) on a yearly basis to retrieve information about the general conditions of the population in the country for planning purposes. The LSMS is aimed at assessing the living conditions of the population in both the urban and rural areas of the country, with a broad set of dimensions. Information is collected once a year. The LIHS is mainly aimed at assessing the work force in the country. It is gathered quarterly and pooled for each administrative year. For the year 2018 both surveys had representativeness at the national and department level, with the former being representative in all departments at both the rural and urban areas and the latter having the same representativeness except for the newly added departments of the Amazonía and Orinoquía regions where data was retrieved only in the urban areas, as most of the population live in the most urbanized areas in those regions. The sampling procedure for both surveys is a) random, b) stratified, c) multistage, and d) clustered.[1] [2]

Index development

The process of index development was as follows[3]: First, the study of redundant variables where indicators that could represent the same notion - aggregated and disaggregated indicator (i.e. housing infrastructure VS housing ceilings, floors and walls) or different alternatives of an indicator (i.e. public services VS access to potable water) - were introduced to an independent PCA to select those that better represented the construct of interest. From those indicators with correlations larger than 0.8, the variable with the highest correlation to the first component was selected for each group of redundant indicators. Second, a subsequent PCA with the remaining set of indicators (redundant and non-redundant) was carried out selecting those with contributions to the first component larger than the average. Third, a final PCA was carried out with the remaining indicators and the first component of this PCA was considered as the initial index. The indicators constitutive of the final PCA were further discussed within the team to explore theoretical validity and an additional PCA was performed incorporating one indicator that was considered relevant for the construct of precarity and that could have been excluded because its contribution was competing with the average contribution of the rest of indicators.

Of note, the selection of indicators within the data sources was based on the different theoretical dimensions of the modes of living and power relations from Breilh's framework[4], but after the first PCA excluding only the redundant indicators per dimension, the following PCAs were performed with all the indicators without compartmentalizing dimensions to allow for their dialectical and interrelated nature.

The appropriateness for conducting a PCA was assessed through the Bartlett's test of sphericity with a significant result ($p<0.001$) and Keiser-Meyer-Olkin (KMO) measure with a value of 0.78.

References

[1] Departamento Administrativo Nacional de Estadísticas (DANE). Ficha metodológica Gran Encuesta Integrada de Hogares. Dirección de Metodología y Producción Estadística/DIMPE. Abr/2016.

[2] Departamento Administrativo Nacional de Estadísticas (DANE). Metodología general Encuesta Nacional de Calidad de Vida-ECV. Dirección de Metodología y Producción Estadística/DIMPE, Abr/2018.

[3] Lalloué B, Monnez JM, Padilla C, Kihal W, Le Meur N, Zmirou-Navier D, et al. A statistical procedure to create a neighborhood socioeconomic index for health inequalities analysis. Int J Equity Health. 2013 Mar 28;12(1):21.

[4] Breilh J. Why Critical Epidemiology? Daring Ethical Science in an Unhealthy Civilization. In: Breilh J, Krieger N, eds. *Critical Epidemiology and the People's Health*. Oxford University Press; 2021:50-110. doi:10.1093/med/9780190492786.003.0003

The citations below correspond to numbers [6, 43, 44, and 48] in the main text.

Supplementary Table S1. Initial set of indicators introduced into the analysis

Dimension	Indicators
**Power relations**	Ethnic communities (Afro-Colombian, Indigenous and Rom communities)
	Lowest social classes (Low and middle low social classes)
	Population younger than 18 years
	Population older than 65 years
	Population older than 65 years living alone
**Metabolic (ecosystem) relations**	Insufficient housing infrastructure
	Overcrowding
	Lack of adequate sewage (Inadequate elimination of sewer waste)
	Absence of home water service
	Flammable cooking fuels
	Solid cooking fuels
	Dwelling affectated by environmental phenomena
	Displacement due to violence (among people that moved from their previous residence in the last years)
**Organization and supports**	Use of government subsidies and supports
	Feeling of insecurity
	Being a victim of robbery or another criminal act
**Culture and spiritual means**	Self-recognition as poor
**Consumption**	Household income lower than the national minimum wage
	Perception of insufficient household income for basic expenses
	Lack of house ownership
	House beneficial owner or de facto occupant
	Digital divide
	Lack of basic supplies
	Lack of car or motorcycle ownership
	Lack of affiliation to the health system
	Not having eaten any of the three main meals of the day
	Not being able to study for lack of money or other responsibilities
	Illiteracy
	School lag (backwardness)
**Work**	Employment instability (Less than 23 months working in the same place)
	Lack of employee benefits and perks
	Partial employee benefits and perks
	No employment contract
	Low satisfaction with job income
	Low job satisfaction
	Inadequate conditions at work
	Unemployed in the labor force
	Lack of social security/Informal work

Supplementary Table S2. Indicators constitutive of the final index of precarity in the modes of living and its operationalization

Dimension	Experience of precarity	Gender disaggregation[a]	Indicator	Numerator	Denominator	Operative definition of composite indicator
**Power relations**	Ethnic communities		Percentage of Afro-Colombian, Raizal, Palenquero(a), Indigenous and Gypsy population	Afro-Colombian, Raizal, Palenquero(a), Indigenous and Gypsy population	Total population	This indicator includes the population considered pertaining to the following ethnic communities according to the National Statistics Office (DANE): Black, Mulatto, Afro-Colombian; Raizal of the San Andrés, Providencia and Santa Catalina archipelago; Palenquero(a); Indigenous and Gypsy (Rom).
	Population under 18 years of age		Percentage of population under 18 years of age	Persons under 18 years of age	Total population	Considers the proportion of the population less than 18 years.
	Population over 65 years of age		Percentage of population over 65 years of age	People over 65 years of age	Total population	Considers the proportion of the population over 65 years.
**Metabolic (ecosystem) Relations**	Cooking with potentially dangerous sources (solid fuels)		Percentage of households that cook with solid fuels	Households that cook with solid fuels	Total households	Households using coal, firewood, wood, charcoal, or waste material for cooking.
	Lack of access to complete home services		Percentage of households without complete access to household public services	Households without complete access to household public services	Total households	A household is considered without complete access to services if it does not have any of these services: Electricity, Water supply or Garbage collection. .
	Overcrowding		Percentage of overcrowded households	Overcrowded households	Total households	Households with three or more people per room are considered overcrowded
	Insufficient housing infrastructure		Percentage of households with insufficient housing infrastructure	Households with insufficient housing conditions in terms of wall materials, roof materials, or floor materials	Total households	A household is considered to have insufficient housing infrastructure when the type of housing or housing materials are deemed inadequate in any of the following areas: roofs, floors, or walls. (**Type of housing**: classified as "Other type of housing," such as a tent, container, wagon, boat, cave, natural shelter, bridge, or similar. **Wall materials**: rough wood planks or boards; prefabricated materials; bamboo, cane, reeds, or other plant-based materials; zinc, coal sacks, metal scraps, plastic, or other waste materials; or no walls. **Floor materials**: earth or sand. **Roof materials**: metal sheets or zinc panels; plastic sheeting; straw, palm leaves, or other plant-based materials; or waste materials.)
	Lack of adequate sewage		Percentage of households with inadequate disposal of excreta	Households with inadequate disposal of excreta	Total households	A lack of adequate sewage is considered for rural areas when the home does not have sewage or the type of sanitary service is a toilet without connection, latrine, toilet with direct discharge to water sources. For urban areas, when the household does not have a sewage system or the type of sanitary service is toilet connected to a septic tank, toilet without connection, latrine, or toilet with direct discharge to water sources.
**Culture and spiritual means**	Self-recognition as poor		Percentage of population who self-recognizes as poor	Population who self-recognizes as poor	Total population	Corresponds to those individuals who answered affirmatively when asked about their self-recognition as poor
**Consumption**	Household income lower than the national minimum wage	X*	Percentage of households with income below the minimum wage for the year 2018	Households with income below the minimum wage for the year 2018	Total households	Corresponds to those households that reported an income less than or equal to $781,242 Colombian pesos
	Digital divide	X	Percentage of households with digital divide	Households with digital divide	Total households	Digital divide is considered when the household does not have internet; Also, if the household has internet, but no computer or cell phone.
	Lack of house ownership	X	Percentage of households without their own home	Households without their own home	Total households	A household is considered to be lacking their own home if the house inhabited is: rented or subleased.
	School lag (backwardness)	X	Percentage of households with school lag	Households with at least one person with school lag	Total households	Households with at least one child or adolescent who are below the national norm of education for their age are considered to have school lag.
	Lack of basic supplies	X	Percentage of households with an absence of complete basic supplies	Households with an absence of complete basic supplies	Total households	Lack of basic supplies is considered when the house lacks at least one of the following assets: washing machine for clothes, refrigerator, electric or gas stove
	Perception of insufficient household income for basic expenses	X*	Percentage of households with insufficient income	Households with insufficient household income	Total households	Corresponds to households where the head of the household perceived that their current income was not enough to cover the minimum expenses
	Illiteracy	X	Proportion of households with illiteracy	Households with at least one illiterate person	Total households	A person aged 15 or over who cannot read and write is considered illiterate.

**Work**	Partial employee benefits and perks	X	Percentage of employed with partial guarantees and benefits	Employed with partial guarantees and benefits	Employed population	Partial benefits are present whenever an employee (with any contract) has only one or two of the following conditions: pay severance, has the right to paid vacations and has the right to premium Christmas. When they have all three conditions the person is considered to have complete benefits.
	Low satisfaction with job/work-activity		Percentage of population with low satisfaction with work or main activity	Dissatisfied with work or main activity	Employed population	Considers the employed population that has low satisfaction with respect to their work.
	Inadequate working conditions (physical, psychological, overload)	X*	Percentage of employed with inadequate working conditions	Employed with inadequate working conditions	Employed population	Inadequate working conditions are considered if at least one of the following conditions is present: poor industrial safety; annoying noise, vibration or shock; extreme temperatures; strong bad smell or presence of dust in the environment; poor lighting or ventilation; insect sting and animal bite; permanent exposure to climatic variations; improper treatment or psychological pressure at work; requires a lot of physical effort; the work demands continuous high intellectual effort; or long working hours (work overload).
	Informal work / Lack of social security	X	Percentage of population in informal work situation	People in informal work situation	Employed population	Considers people without pension scheme or occupational risk insurance

[a] Indicators were disaggregated based on the head of household, and a sensitivity analysis was conducted, including disaggregated indicators. The results of the Principal Component Analysis (PCA) using the disaggregated indicators yielded the same findings as those using the aggregated indicators (i.e., both indicators for men and women were retained for all the respective indicators selected in the overall index).

* While most indicators showed a similar proportion by gender, slight differences in the mean proportions of certain indicators were identified, as noted below:

- Mean proportion of households with household income lower than the national minimum wage: men 34%; women 40%
- Mean proportion of households with a perception of insufficient household income for basic expenses: men 43%; women 47%
- Mean proportion of employed with inadequate working conditions (physical, psychological, overload): men 65%; women 41%

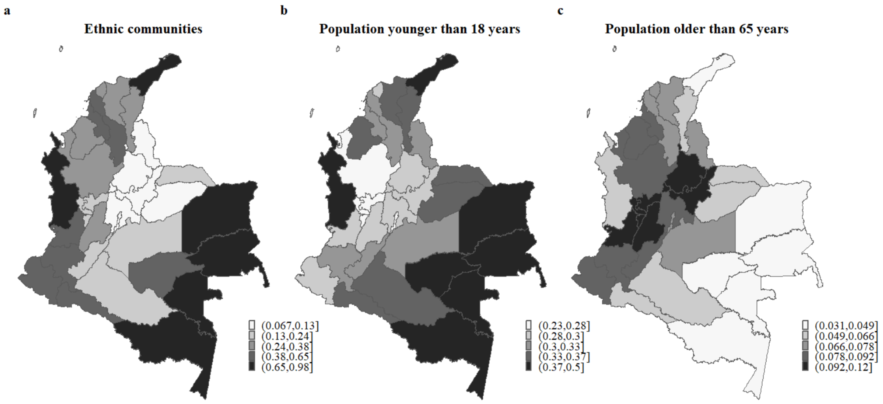

Supplementary Figure S1. Geographical representation of indicators of power relations. Maps represent the proportion of population with a given characteristic by department: a. Ethnic communities (Afro-Colombian, Indigenous, and Rom communities), b. Population younger than 18 years, and c. Population older than 65 years.

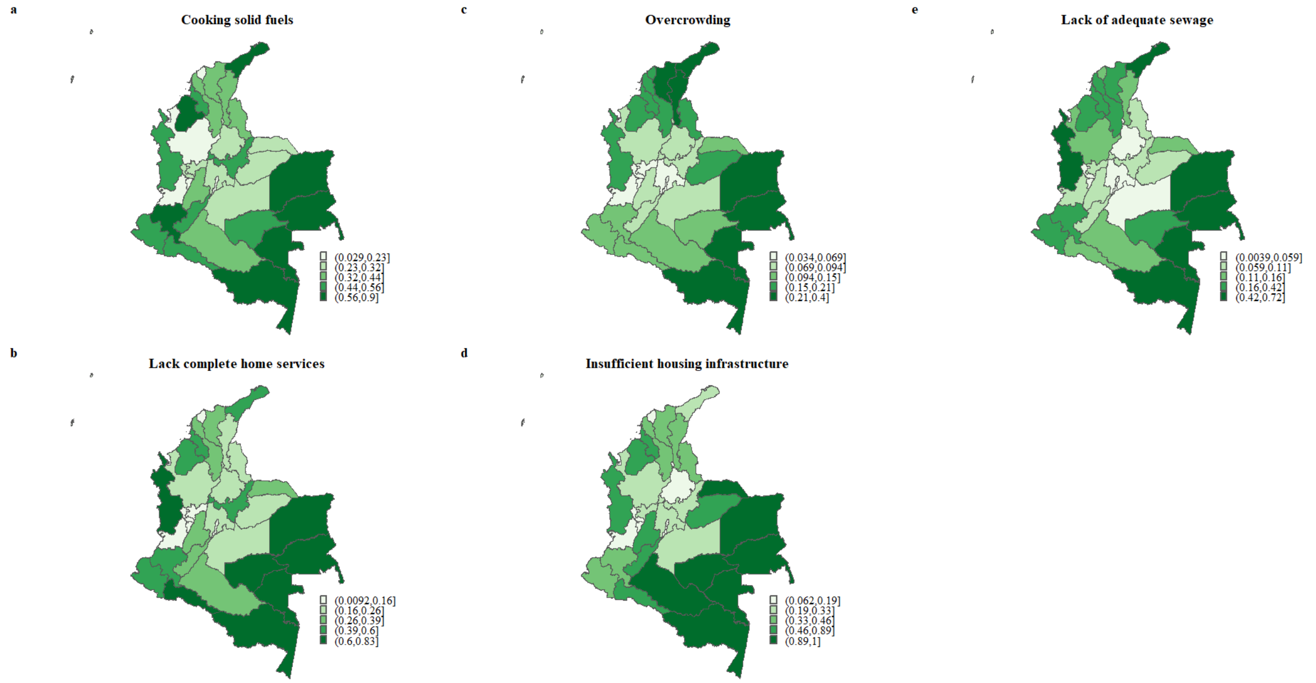

Supplementary Figure S2. Geographical representation of indicators from the metabolic (ecosystem) relations dimension. Maps represent the proportion of the population with a given characteristic by department: a. Cooking with potentially dangerous sources (solid fuels), b. Lack of complete home services, c. Overcrowding, d. Insufficient housing infrastructure, e. Lack of adequate sewage.

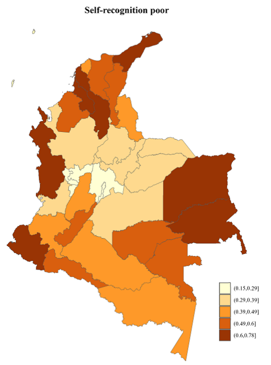

Supplementary Figure S3. Geographical representation of indicators from the culture and spiritual means dimension. The map represents the proportion of population self-recognized as poor.

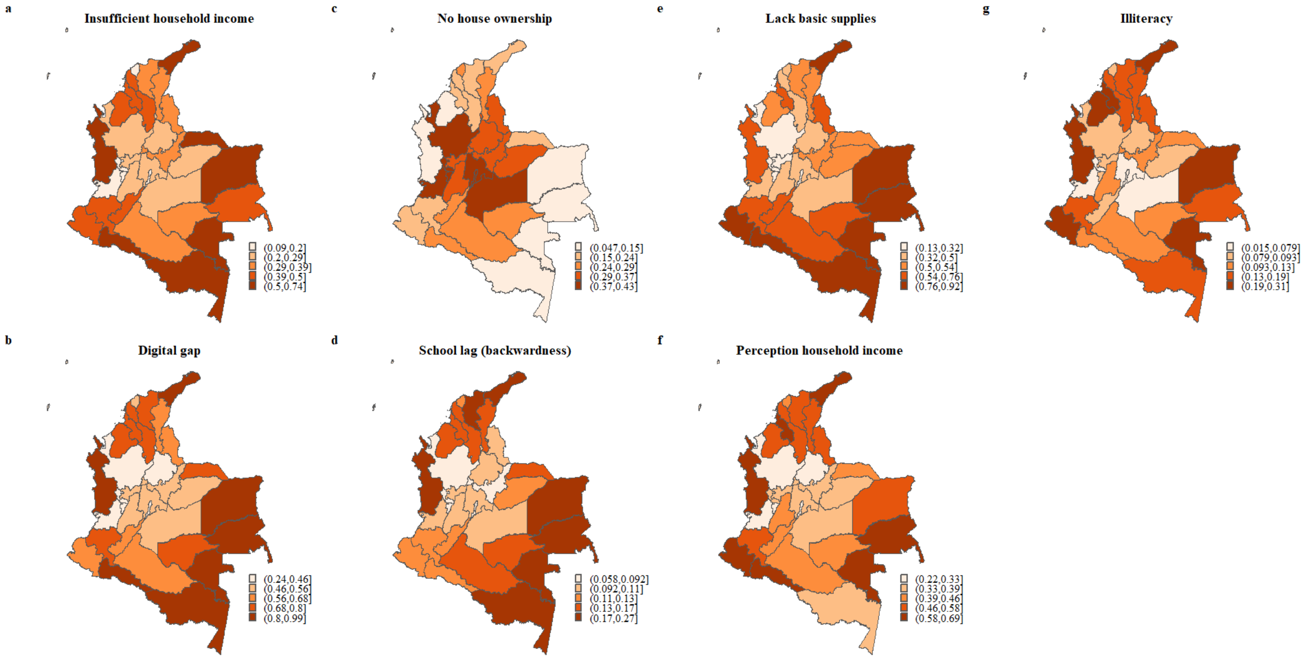

Supplementary Figure S4. Geographical representation of indicators from the consumption dimension. Maps represent the proportion of population with a given characteristic by department: a. Insufficient household income (household income lower than the national minimum wage), b. Digital divide, c. Lack of house ownership, d. School lag (backwardness), e. Lack of basic supplies, f. Perception of insufficient household income for basic expenses, g. Illiteracy.

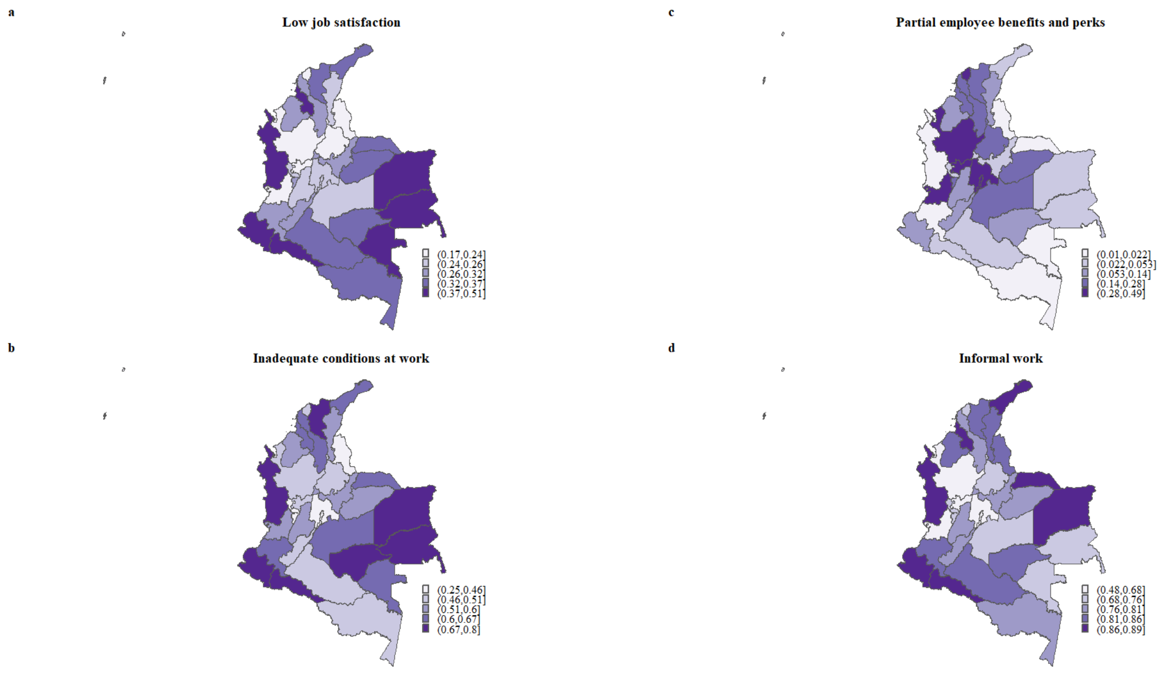

Supplementary Figure S5. Geographical representation of indicators from the work dimension. Maps represent the proportion of population with a given characteristic by department: a. Low job satisfaction, b. Inadequate conditions at work, c. Partial employee benefits and perks, d. Lack of social security/Informal work.

Section 2. Spatial Autocorrelation analyses

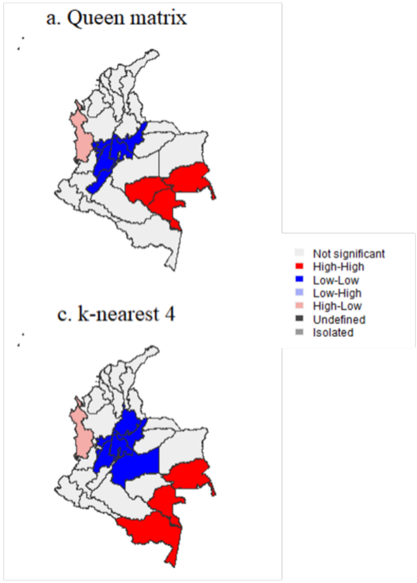

Supplementary Figure S6. Results of the Local Indicators of Spatial Association (LISA) on the index of precarity using different spatial weights matrices. Maps show the local clusters from the index of precarity: In red clusters of high values surrounded by high values (high-high); in blue clusters of low values surrounded by low values; in light red an outlier of a high value surrounded by low values. In light grey non-significant values (not significant). Different spatial matrices were defined: a. Queen, b. Rook, c. 4 k-nearest neighbors, d. Adaptive kernel with 4 neighbors. The islands of San Andrés and Providencia were classified as non-significant through the four spatial matrices.

Section 3. Regressions with health indicators

Supplementary Table S3. Results from a negative binomial regression model assessing the association of the precarity index and under five and infant deaths, pooled sample 2017-2019

	SMR	95%CI	p
**Negative binomial model**			
Under five deaths	1.19*	[1.08,1.31]	0.001
Infant deaths	1.12	[1.00,1.26]	0.042

*p<0.05

Supplementary Table S4. Results from spatial lag x regression models assessing the association of the precarity index and under five and infant mortality rates, pooled sample 2017-2019

	Estimate	Std. Error	p
**U5MR**			
Precarity index	12.55	3.94	0.003*
Lagged precarity index	-5.17	6.62	0.44
**Infant mortality rate**			
Precarity index	1.84	0.91	0.05
Lagged precarity index	-0.96	1.52	0.53

*p<0.05
